# Genome-Wide Association Studies of Quantitatively Measured Skin, Hair, and Eye Pigmentation in Four European Populations

**DOI:** 10.1371/journal.pone.0048294

**Published:** 2012-10-31

**Authors:** Sophie I. Candille, Devin M. Absher, Sandra Beleza, Marc Bauchet, Brian McEvoy, Nanibaa’ A. Garrison, Jun Z. Li, Richard M. Myers, Gregory S. Barsh, Hua Tang, Mark D. Shriver

**Affiliations:** 1 Department of Genetics, Stanford University, Stanford, California, United States of America; 2 HudsonAlpha Institute for Biotechnology, Huntsville, Alabama, United States of America; 3 Department of Anthropology, The Pennsylvania State University, University Park, Pennsylvania, United States of America; 4 Smurfit Institute of Genetics, Trinity College Dublin, Dublin, Ireland; 5 Department of Human Genetics, University of Michigan, Ann Arbor, Michigan, United States of America; University of Bristol, United Kingdom

## Abstract

Pigmentation of the skin, hair, and eyes varies both within and between human populations. Identifying the genes and alleles underlying this variation has been the goal of many candidate gene and several genome-wide association studies (GWAS). Most GWAS for pigmentary traits to date have been based on subjective phenotypes using categorical scales. But skin, hair, and eye pigmentation vary continuously. Here, we seek to characterize quantitative variation in these traits objectively and accurately and to determine their genetic basis. Objective and quantitative measures of skin, hair, and eye color were made using reflectance or digital spectroscopy in Europeans from Ireland, Poland, Italy, and Portugal. A GWAS was conducted for the three quantitative pigmentation phenotypes in 176 women across 313,763 SNP loci, and replication of the most significant associations was attempted in a sample of 294 European men and women from the same countries. We find that the pigmentation phenotypes are highly stratified along axes of European genetic differentiation. The country of sampling explains approximately 35% of the variation in skin pigmentation, 31% of the variation in hair pigmentation, and 40% of the variation in eye pigmentation. All three quantitative phenotypes are correlated with each other. In our two-stage association study, we reproduce the association of rs1667394 at the *OCA2/HERC2* locus with eye color but we do not identify new genetic determinants of skin and hair pigmentation supporting the lack of major genes affecting skin and hair color variation within Europe and suggesting that not only careful phenotyping but also larger cohorts are required to understand the genetic architecture of these complex quantitative traits. Interestingly, we also see that in each of these four populations, men are more lightly pigmented in the unexposed skin of the inner arm than women, a fact that is underappreciated and may vary across the world.

## Introduction

Human pigmentation of the skin, hair, and eyes varies worldwide. Skin pigmentation forms a gradient correlated with latitude, and variation in hair and eye color is particularly extensive in Europe (reviewed in [1]). For skin color, global-level variation is likely driven primarily by natural (ecological) selection in response to UV radiation levels. The strong linear relationship between global skin pigmentation variation and latitude/UV radiation levels is thought to result from UV-mediated vitamin D synthesis, folate photolysis, sunburn, and skin cancer [2]–[5]. Unlike for global skin pigmentation, there is no obvious ecological selective pressure to explain the variation of hair and eye color or local variation in skin pigmentation. Given the conspicuousness of hair, eye, and skin color, a reasonable hypothesis is that variation in these traits has been shaped by sexual selection [6], 7. Because the geographical differentiation of these traits correlates with the demographic history of human populations, a strong and intrinsic confounding with population structure presents a significant challenge for genetic association studies of human pigmentary traits.

Genome-wide association studies (GWAS) have proven to be useful for identifying genes for hair and eye color variation in populations of European ancestry in part because of methods to correct for population stratification [8]–[13]. Complementing earlier candidate genes studies (for example, those that lead to the demonstration of an important role for *MC1R* in hair color), these GWAS have provided a growing list of genes that reproducibly contribute to variation in hair and eye color. Phenotypic variation between blue and non-blue eye color is explained in large part by genetic variation at the *OCA2/HERC2* locus along with genetic variation at *SLC45A2, SLC24A4*, *IRF4*, *TYR*, and *TYRP1*; between blond and brown hair color by variation at *OCA2/HERC2*, *SLC45A2*, *SLC24A4*, *IRF4/EXOC2*, *TPCN2*, *KITLG*, and *MC1R*; between red and non-red hair color by variation at *MC1R* and *ASIP* (reviewed in [14]). This work has enabled the development of prediction models for categorical eye and hair color based on SNP genotypes that have good accuracy [15], [16]. Nonetheless, while these loci explain a large fraction of the phenotypic variance in Europeans, approximately 20–30% for black to blond hair color and 40–60% for eye color, the remaining phenotypic variation in these traits remains unexplained [8], [9], [11].

Genetic studies of hair and eye color are made easier by the fact that these traits appear to fall into subjective categories such as red/blond/brown/black for hair color and blue/green/brown for eye color. These phenotypes can be easily observed or even self-reported and have been used as such in most GWAS. Skin color is less amenable than hair or eye color to subjective categorization and self-report because skin color is perhaps both less categorical in distribution and less accurately described in words. One GWAS of South Asians quantified this phenotype by measuring the skin reflectance and performed a genome scan for subjects falling in the 20% tails of the skin pigmentation distribution [17]. In this study, three genes, *SLC24A5*, *SLC45A2*, and *TYR* were found to explain a large fraction of the pigmentation difference among individuals with high and low skin reflectance. To our knowledge, no other GWAS has attempted to map skin color although some have used the related subjective traits, such as namely skin sensitivity to sun, the ease of tanning, or the amount of freckling [8], [12], [13].

Skin, hair, and eye color are in fact all three quantitative traits, and their true distributions are only approximated by categories. The use of categories necessarily results in a loss of information, leading to a loss of statistical power to detect genetic effects. This was demonstrated in one study of eye color by Liu et al. [11] who obtained both subjective categorical measures of eye color and objectively quantified eye color from digital photographs. These authors report that the quantitation improved the power to detect the association signals at some known pigmentation genes and also facilitated the identification of two novel replicated eye color loci at 17q25 and 21q22.

To more comprehensively characterize skin, hair, and eye pigmentation in Europeans, we obtained both subjective categorical and objective quantitative measurements of skin, hair, and eye color in participants from four European countries: Ireland, Poland, Italy, and Portugal. These countries, located near the geographical extremes of Europe, were chosen to capture a large fraction of European phenotypic variation. We studied the distributions and the correlations of the quantitative pigmentation phenotypes within and among these European countries and between the sexes, compared them to subjective self-assessments of pigmentation, and conducted a GWAS for each quantitative trait.

## Materials and Methods

### Subjects

Research participants were recruited in four European cities: Dublin (Ireland), Warsaw (Poland), Rome (Italy), and Porto (Portugal). In Dublin and Rome, participant recruitment was conducted at a university, while in Warsaw and Porto, the sampling took place in two locations, at a university and a research institute. Participants completed a questionnaire that included a self-report of their hair and eye color, their height, and the ancestry of their four grandparents.

### Ethics Statement

Written informed consent was obtained from each participant and the study was approved by the Institutional Review Boards or ethics committees of The Pennsylvania State University, Stanford University, University of Porto, Trinity College Dublin, and Wojskowy Instytut Medyczny in Warsaw.

### Phenotype Collection and Analysis

Skin and hair pigmentation were measured as the melanin (M) index by reflectance spectroscopy using the DermaSpectrometer (Cortex Technologies, Denmark). The M index is defined as 100×log_10_ (1/proportion red reflectance at 655nm) [18]. For skin pigmentation, three M index measurements were made on the medial aspect of each upper arm. These six measurements were averaged for each participant. The inner upper arm was chosen as a site of sampling to avoid as much as possible confounding by variable sun exposure and variability in tanning ability. For hair pigmentation, three measures of the M index were made near the crown of the head and were averaged. Only participants who reported having natural hair color (*i.e.* not dyed) were measured. Higher M indexes correspond to darker pigmentation for both skin and hair pigmentation. For eye pigmentation, the iris color score (C) was calculated from the analysis of digital photographs taken under controlled light conditions using a cardboard box with form fitting foam padding such that the only illumination was from the camera flash. To help ensure that the camera flash was consistently charged, all photos were taken with the camera connected to the AC/DC power supply and not using battery power. The C score combines the luminance and the red, green, and blue color reflectance of the iris into one number [19]. We transformed the C score into C’ = C_max_-C (C_max_ = 3.741 in our data) so that higher C’ values correspond to darker irises. Summary statistics for the skin and hair M index and the eye C’ score are reported in Table 1.

**Table 1:** Skin, hair, and eye pigmentation by sex and country.

	Skin (M)				Hair (M)				Eye (C')			
	Males		Females		Males		Females		Males		Females	
	Mean (sd)	n	Mean (sd)	n	Mean (sd)	n	Mean (sd)	n	Mean (sd)	n	Mean (sd)	n
Ireland	25.8 (2.4)	59	27.0 (2.0)	87	106.7 (21.0)	55	96.3 (17.8)	48	1.07 (0.58)	58	1.09 (0.62)	88
Poland	28.5 (2.0)	20	30.4 (1.9)	52	109.5 (14.9)	18	107.5 (19.2)	25	1.44 (0.63)	20	1.37 (0.71)	52
Italy	30.0 (2.5)	29	31.3 (2.0)	80	132.4 (12.3)	25	128.2 (18.3)	50	2.11 (0.41)	29	1.95 (0.56)	79
Portugal	28.3 (2.7)	45	30.3 (2.5)	97	125.8 (14.0)	43	123.6 (15.9)	77	2.14 (0.36)	45	2.04 (0.40)	97

We also recorded the self-reported hair and eye colors of the participants. After excluding data from individuals who reported more than one color, hair colors were grouped into the following four categories and given a score of 1 to 4, respectively: red (n = 6), blond (n = 58), brown (n = 252) and black (n = 11). For eye color, self-reported colors were grouped into the following four colors and given a score 1 to 4, respectively: blue (blue and blue-gray, n = 123), green (n = 97), hazel (n = 14) and brown (n = 212).

To compare phenotypes among countries and between the sexes, we used a linear model in R (http://www.R-project.org/). The percent of phenotypic variance accounted for by different factors is r^2^, the coefficient of determination in a linear regression calculated as: variance(fitted phenotype values)/variance(phenotype).

### Genotyping and Quality Control in the Genome-wide Association Study

DNA was extracted from blood from finger stick collected on FTA paper (Whatman Inc., Clifton, NJ). The genotypes of 180 European women were obtained at 317,503 single nucleotide polymorphisms (SNPs) using the HumanHap300v1 BeadChip (Illumina, Inc., San Diego, CA). For quality control, two participants were genotyped in duplicate and showed good concordance of the genotypes (99.97% on average). SNPs with a minor allele frequency (maf) <1% or >10% missing rate were excluded from the analysis. One individual with >10% missing data and three individuals that were genetic outliers in the population structure analyses were also excluded from all analyses. In total, 313,763 SNPs were analyzed in 176 individuals for the genome-wide association study (GWAS).

To detect potentially poorly genotyped SNPs, an exact test of deviation from Hardy-Weinberg equilibrium (HWE) was implemented for each SNP in the four population samples. There were 186 SNPs that had a p-value <0.01 in two or more populations, none of which were part of the replication set.

### Population Structure Analysis in the GWAS

Of the 45 women recruited in each of Ireland, Italy, Poland, and Portugal and chosen for inclusion in the GWAS, 38, 39, 41 and 40, respectively, reported that their four grandparents had ancestry in the country of sampling. To determine whether any individuals were genetic outliers within each sampling site, a distance to the nearest neighbor analysis was conducted in PLINK (–neighbour option) (http://pngu.mgh.harvard.edu/purcell/plink/, [20]). Within each country, a measure of similarity in terms of identity by state between each individual and their nearest neighbor was calculated and transformed into a z score. Z score distributions were examined for the first to the fifth neighbor. Three individuals, one recruited in Ireland and two in Portugal, were clearly outliers within their group (z score <−3 for first to fifth nearest neighbor). These individuals were removed from all association testing.

Population structure was also examined by principal components analysis (PCA) performed in SMARTPCA [21]. Principal components were calculated based on a set of 263,607 SNPs that had been subjected to higher quality control than the SNPs considered for the GWAS. Specifically, this set excludes SNPs with maf <0.05, missing rate >2%, HWE deviation p-value <0.01 in at least two populations. Also excluded were SNPs on the X and Y chromosomes. The first PCA run identified the same three outliers as the PLINK neighbor analysis (number of standard deviations exceeded along one of the first 10 components >6). These three persons were excluded from the final PCA. The first 3 PCs explain 1.02%, 0.74% and 0.65% of the variance in the genotypes.

### Genotyping and Quality Control in the Replication Stage

Replication of the most significant GWAS association signals was attempted in 294 individuals (104 Irish, 27 Polish, 64 Italian, and 99 Portuguese; 153 males and 141 females) using a custom designed GoldenGate assay (Illumina, Inc., San Diego, CA). Twenty-six, 33, and 44 SNPs with p-values <10^−4^ in the GWAS were selected for replication of the skin, hair, and eye color associations, respectively. SNP rs17160255 failed the GoldenGate assay design stage (Illumina Designability Rank of 0) and was replaced by rs17160261, a SNP in high linkage disequilibrium (D’ = 1 and r^2^ = 0.94 in Utah residents with ancestry from northern and western Europe [CEU], HapMap release 27, http://hapmap.ncbi.nlm.nih.gov). SNPs with missing rate >10%, maf <0.01, and p-values<0.01 in at least two populations for a test of deviation from HWE were excluded. Two SNPs on chromosome X with heterozygous genotypes in males were also excluded. Based on these quality control criteria, genotypes were obtained for 21, 26, and 34 of the SNPs selected from the results of the skin, hair, and eye pigmentation GWAS respectively. The Pearson correlation between all-sample allele frequency in the GWAS and replication samples was high (r = 0.97).

### Statistical Analyses of the GWAS and the Replication

Statistical analyses were performed in PLINK or R. For the GWAS, the replication, and the replication/GWAS combined analysis, the association between the quantitative phenotype and SNP genotype was tested using a linear model, where each copy of the minor allele was assumed to have the same additive effect on the phenotype. Population structure was corrected for by including as covariates in the linear model three dummy variables that code for the four countries of sampling (see below for the alternative method of using PC scores). In the replication and the replication/GWAS combined analysis, we additionally adjusted for sex. Age was not correlated with any of the pigmentation traits most likely due to the narrow age range in our study: mean ages (standard deviation) were 21 (3.2), 24 (4.5), 23 (3.1), and 25 (4.2) for the Irish, Polish, Italian, and Portuguese participants, respectively. Results from the GWAS were plotted as Manhattan plots in Haploview [22].

In the GWAS analysis, to assess the robustness of the p-values derived from the linear model, empirical p-values were also obtained by permuting the phenotypes within each country of sampling 10,000,000 times for all GWAS association signals with linear model p-value<10^−4^. The p-values obtained by this permutation method account for population structure and do not rely on the distributional assumptions of the linear model. The p-values from permutations for these GWAS association signals (with a linear model p-value <10^−4^) were all smaller than 10^−3^. The permuted p-values for the top hits of the skin (rs9809315), hair (rs262825), and eye pigmentation (rs1667394) GWAS were 1×10^−7^, 6.3×10^−6^, and <1×10^−7^, respectively. They are similar to those obtained using the linear model.

We compared the GWAS p-values corrected for population structure by country of sampling with those corrected by using the first three genetic PCs. Again, the results were similar. The Pearson correlation coefficients between the linear model t-statistics from the two GWAS results are 0.96, 0.97, and 0.96 for the skin, hair, and eye pigmentation scans, respectively. Although the most significant p-values show some fluctuation, they are consistent. For skin pigmentation, the two most significant SNPs in PC-based analysis, rs9809315 (p = 3.5×10^−6^) and rs6664692 (p = 3.1×10^−6^), are the first and ninth most significant SNPs when population structure is corrected by country of sampling. For hair pigmentation, the most significant SNP in PC-based analysis is rs7712713 (p = 1×10^−5^), which when using the country of origin also had a p-value of 1×10^−5^. For eye pigmentation, the most significant SNP for both is rs1667394 (p = 1.8×10^−9^). A quality control measure for the association testing, the genomic control inflation factor (lambda) [23], was calculated for each GWAS and Q-Q plots were drawn (Figure S1). The genomic control lambda factors for the skin, hair and eye pigmentation GWAS were each close to 1 and there was no systematic deviation from expectation (the diagonal) in the Q-Q plots.

Genotypes of the GWAS samples at HapMap 2 SNPs were imputed in a 2.5 Mb window including the *OCA2/HERC2* locus. The software MACH was used to perform the imputation based on the phased HapMap 2 release 21 genotypes of CEU [24], [25]. Power calculations were performed using the Genetic Power Calculator (http://pngu.mgh.harvard.edu/~purcell/gpc) [26].

## Results

### Quantitative Skin, Hair and Eye Pigmentation Phenotypes

**Figure 1:**
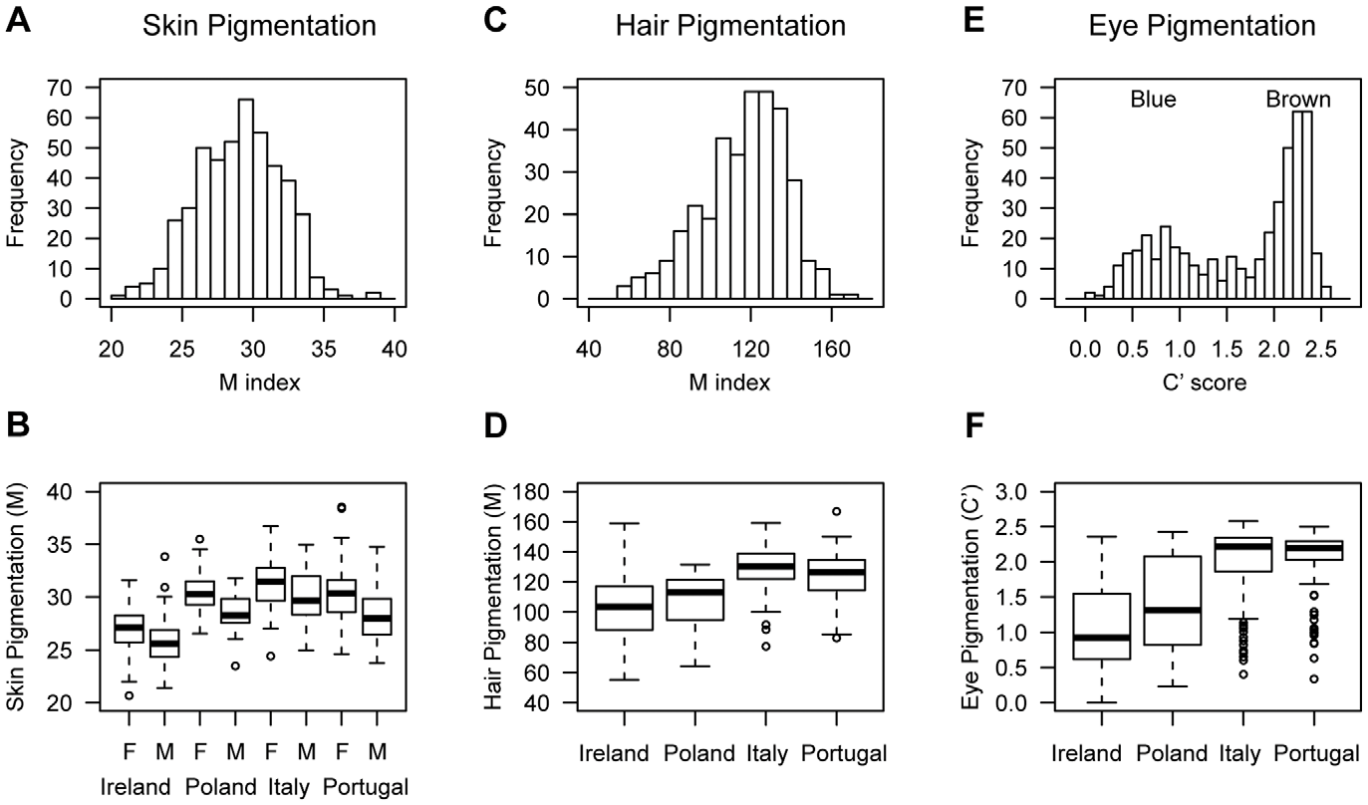
Distribution of skin, hair, and eye pigmentation. Skin pigmentation histogram (**A**) and boxplot by country of sampling and sex (**B**) in 469 individuals showing the normality of the phenotype distribution and the differences between sexes and among countries. Males (M) have consistently lighter pigmentation (lower scored) than females (F) in all four countries. Among countries, the largest pigmentation difference is with Ireland, where, in our sample, individuals have lighter pigmentation or lower M index on average than in Poland, Italy, or Portugal. Hair pigmentation histogram (**C**) and boxplot by country (**D**) in 341 individuals showing the distribution of hair pigmentation and the differences among countries. In our sample, individuals from Northern European countries (Ireland, Poland) have on average lighter hair pigmentation than individuals from Southern European countries (Italy, Portugal). The distributions in males are similar to those in females in all countries except Ireland, where, in our sample, males have darker hair color than females (not shown). Eye pigmentation histogram (**E**) and boxplot by country (**F**) in 468 individuals showing the bimodal distribution of eye pigmentation and the differences among countries. Comparison with self-reported phenotypes shows that the two modes of the distribution correspond to blue and brown eye color, while individuals reporting green and hazel eye color have intermediate C’ values. As with hair pigmentation, in our sample, individuals from Northern European countries have on average lighter eye pigmentation than individuals from Southern European countries.

Quantitative measures of skin, hair, and eye pigmentation were obtained for 470 individuals recruited in Ireland, Poland, Italy, and Portugal and included in a two-stage genetic association study of pigmentary phenotypes (Table 1). While the phenotype distributions are largely overlapping among countries and between the sexes (Figure 1), there are some significant average differences (Table S1).

The skin M index has a normal distribution (Figure 1A, Shapiro-Wilk test of normality p = 0.5, n = 469) and it differs between sexes and among countries (Figure 1B). Although some conflicting data exists, studies of sexual dimorphism in skin color generally have revealed that females tend to be more lightly pigmented than males (reviewed in [2], [27]). Surprisingly, we find in our cohort that males have lighter skin pigmentation (lower M) than females in all four European countries. The average sex differences in skin M index and 95% confidence interval (CI) are 1.24 M index units (95% CI = 0.52–1.95, p = 8×10^−4^), 1.91 M index units (95% CI = 0.92–2.91, p = 3×10^−4^), 1.32 M index units (95% CI = 0.38–2.26, p = 6×10^−3^), and 2.04 M index units (95% CI = 1.13–2.95, p = 2×10^−5^), in Ireland, Poland, Italy, and Portugal, respectively. In the combined sample, after adjusting for country of sampling, this sex difference is highly significant: males are on average more lightly pigmented than females by 1.57 M index units (95% CI = 1.14–2.01, p = 4×10^−12^). With the exception of the Poland vs. Portugal comparison, average skin pigmentation is also different among countries. Most significant is the difference in skin pigmentation between Ireland and the other countries: Irish participants have lighter skin pigmentation than Polish, Italian, and Portuguese participants (p<2×10^−16^ for all three comparisons, Table 1 and Table S1).

The hair M index has a unimodal distribution skewed to the left, at smaller M index or lighter pigmentation (Figure 1C, n = 341). It differs between sexes in only one country, Ireland, where males have darker hair color than females (p = 8×10^−3^, Table 1 and Table S1). While hair pigmentation differs among all countries, it is most differentiated between Northern and Southern Europe (Figure 1D). Hair pigmentation is lighter in the Northern European countries, Ireland and Poland, than in the Southern European countries, Italy and Portugal (p<10^−7^ for all four comparisons, Table 1 and Table S1). Similarly to skin pigmentation, Irish participants have the lightest hair pigmentation of all groups.

The C’ score for eye pigmentation has a bimodal distribution (Figure 1E, n = 468). There is no evidence of a difference in C’ between males and females but average C’ differs among countries, with the exception of the Italy vs. Portugal comparison. As with hair pigmentation, the greatest difference in eye pigmentation occurs between the Northern and Southern European countries. In our sample, Irish and Polish participants have lighter eye pigmentation than Italian and Portuguese people (p<10^−9^ for all four comparisons, Table 1 and Table S1). Like skin and hair pigmentation, Irish participants have the lightest eye pigmentation on average (Figure 1F, Table 1).

### Comparison of Quantified and Self-reported Phenotypes

**Figure 2:**
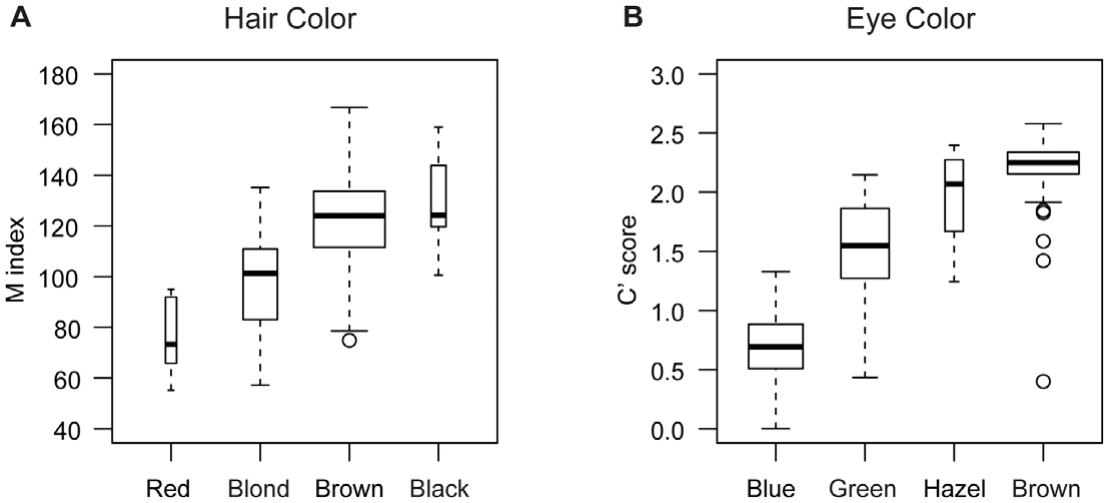
Comparison of measured and self-reported hair and eye color. (**A**) The boxplots of hair pigmentation (M index) binned by self-reported hair color show that there is a good correspondence between categorical hair color and the M index. On average, individuals with red hair color (n = 6) have lower M indices than blond individuals (n = 58). Individuals reporting brown (n = 252) or black (n = 11) hair color have the highest M indices. (**B**) Similarly for eye pigmentation, the C’ score corresponds well to the self-reported eye color. Individuals reporting blue (n = 123), green (n = 97), hazel (n = 14), and brown (n = 212) eye color have increasing average C’ scores. We note that one individual has a discrepant self-reported color vs. C’ score. This individual self-reported light brown eye color but the C’ score is indicative of most likely blue eye color. This individual was part of the replication cohort and removing the sample from the association study produces similar p-values, only slightly less significant for the *OCA2/HERC2* SNPs. Each box in the plot is drawn with a width proportional to the square root of the sample size.

For hair and eye color, we compared the M and C’ measurements with the usual categories of hair and eye color, namely: red, blond, brown, and black hair color, and blue, green, hazel, and brown eye color, obtained through self-report. A score of 1 to 4 was assigned to each increasingly darker category for both self-reported hair (red to black) and eye color (blue to brown). Self-reported phenotypic scores and quantified phenotypes are highly correlated (Figure 2). The Pearson correlation between the hair M index and hair color score is 0.52 and the correlation between the eye C’ score and the eye color score is 0.89. For hair color, individuals reporting red hair color have the lowest median M index (median M = 73), followed by blond (median M = 101) and then brown or black (median M = 124). For eye pigmentation, the comparison of the C’ score and the self-reported eye color shows that the two modes of the C’ distribution correspond to blue and brown eye color, while green and hazel eye color correspond to intermediate C’ scores. The median C’ scores for blue, green, hazel, and brown self-reported eye colors were 0.69, 1.55, 2.07, and 2.25, respectively.

### Stratification of the Phenotypes

The three pigmentary phenotypes are highly stratified across European countries. Country of origin explains 35.3%, 30.8%, and 39.7% of the variation in the skin, hair, and eye pigmentation, respectively. By comparison, pigmentation phenotypes are much less stratified by sex, explaining only 10.3%, 0.0%, and 0.0% of the phenotypic variance in skin, hair, and eye pigmentation, respectively. It is interesting to contrast the stratification of pigmentary traits to that of height, a classical example of phenotypic stratification in Europe. We find that height is much less stratified by country than pigmentary traits but much more stratified by sex: country explains only 4.9% of the variance in height, while sex explains 45.8% of the variance in height. In conclusion, in this European sample, pigmentation phenotypes are mainly stratified by country, whereas height is mainly stratified by sex.

### Correlations between Phenotypes

Considering all four countries together, and only adjusting for sex, the correlations between pigmentation phenotypes are high (Pearson r = 0.40, p<2×10^−16^ for skin and eye, Pearson r = 0.47, p<2×10^−16^ for hair and eye, Pearson r = 0.42, p = 1×10^−15^ for hair and skin). European individuals with lighter skin pigmentation tend to have lighter hair and eye pigmentation. This correlation is explained in part, but not completely, by population structure since the same trend is also found after adjusting for mean differences among countries, albeit the correlations are attenuated: skin and eye (Pearson r = 0.13, p = 5×10^−3^), hair and eye (Pearson r = 0.15, p = 7×10^−3^), and hair and skin pigmentation (Pearson r = 0.18, p = 7×10^−4^) are still correlated in the four populations combined, after adjusting for both country and sex. Within each country the results are variable. Skin and eye pigmentation are correlated in Ireland. Hair and eye pigmentation are correlated in Portugal. Skin and hair pigmentation are correlated in Poland and Italy (Table S2).

### Population Structure of the GWAS Samples

A two-stage association study was performed to identify the genetic variants that contribute to pigmentation variation in Europe. In the first-stage, 180 females, 45 recruited in each of four countries, were genotyped at 317,503 SNPs. After genotyping quality control, 179 females and 313,763 SNPs were considered in subsequent analyses.

**Figure 3:**
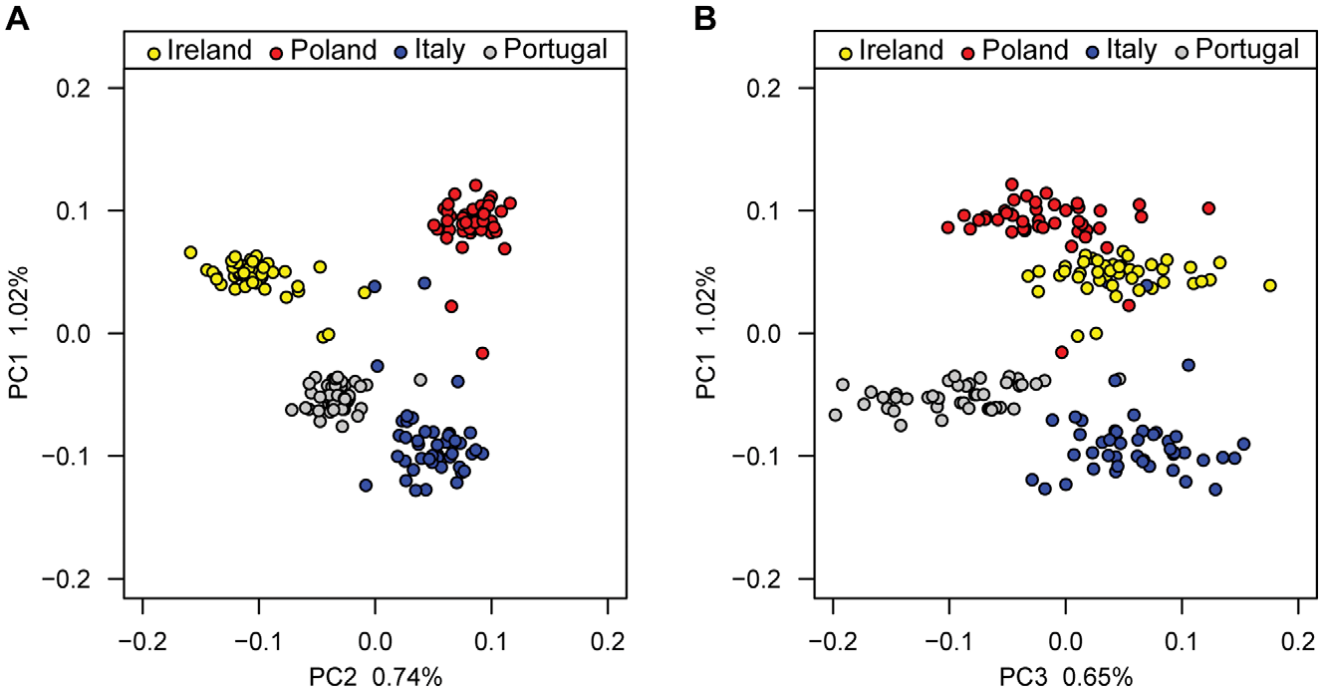
Population structure of the GWAS samples. (**A**) Plots of GWAS individuals on genetic PC1 and PC2 show that individuals largely cluster by country of sampling. PC1 divides the samples according to a North/South geographical axis, while PC2 divides the samples along an East/West geographical axis. Individuals from Ireland, Poland, Italy, and Portugal are colored in yellow, red, blue, and gray, respectively. (**B**) The plot of individuals on PC1 and PC3 shows that individuals from Portugal tend to have lower values on PC3 than individuals from Italy and Ireland, while individuals from Poland have intermediate values on PC3.

PCA was performed to characterize the population structure of our cohort. Three individuals, one with Russian self-reported ancestry and two Portuguese with ancestry in India and Africa, were identified as outliers by PCA. After removing these, 176 individuals were considered for the subsequent analyses. The first two principal components (PCs) together separate the individuals into the four countries of sampling (Figure 3A). PC1 separates Northern (Poland, Ireland) from Southern (Italy, Portugal) countries. PC2 separates Eastern (Poland, Italy) from Western (Ireland, Portugal) countries. On the PC1 by PC2 plot, most participants cluster by country of sampling while ten individuals are found between the clusters formed by each country. We note that these individuals had reported either mixed European ancestry or ancestry from other European countries than where they were sampled. The alignment of the first two genetic PCs with geography and the clustering of individuals by European country are consistent with the results of a previous larger study of European genetic structure [28]. Finally, we find that PC3 separates Portugal from Ireland and Italy, while Poland has more intermediary values on PC3 (Figure 3B). In order to ensure that samples were genetically homogeneous within each of the four sampling sites, we also performed nearest neighbor analyses in terms of identity by state in PLINK. The same three outliers as in the PCA were identified. No other outliers were detected.

### Population Structure Correction

**Figure 4:**
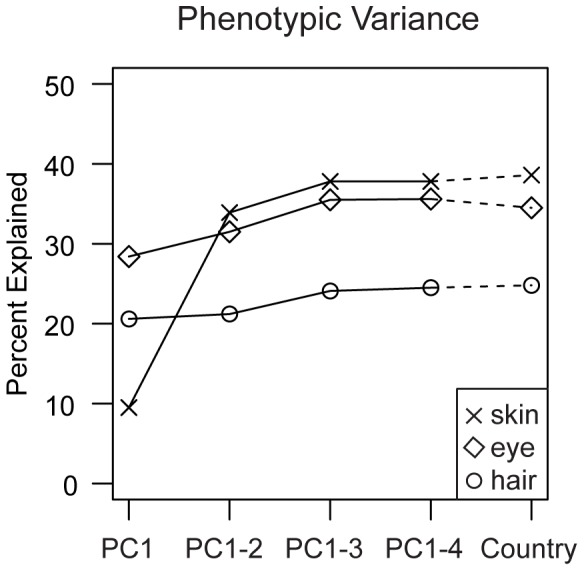
Percent phenotypic variance explained by increasing numbers of genetic PCs or country of sampling. Plotted is the percent phenotypic variance explained by genetic PC1 (PC1); PC1 and PC2 (PC1–2); PC1, PC2, and PC3 (PC1–3); PC1, PC2, PC3, and PC4 (PC1–4); or country of sampling (country) in the GWAS cohort. The first three genetic PCs, similar to the country of sampling, explain a remarkably large proportion of the variation in pigmentation: 38.6/37.8%, 24.8/24.1%, 34.5/35.5% for the skin, hair, and eye pigmentation phenotypes by country of sampling/3 PCs. Including PC4 does not explain significantly more phenotypic variation.

We considered two possible correction methods for the population structure in the GWAS: a correction based on the country of sampling and a correction based on genotype-derived PCs. We first compared the percent of phenotypic variance explained by genetic PCs or by country of sampling. For all three pigmentation phenotypes, the percent variance explained increases as the number of PCs used in the linear model increases to three and then stabilizes (Figure 4). In the GWAS cohort, the first three PCs together account for 37.8%, 24.1%, and 35.5% of the variation in skin, hair, and eye pigmentation respectively. Country of sampling explains a similarly large fraction of variation in the phenotypes of the same samples: 38.6%, 24.8% and 34.5% of the variation in skin, hair, and eye pigmentation respectively. This observation, combined with the clustering of individuals in the PCA by country and the genetic homogeneity within each country revealed by the PLINK neighbor analysis, motivated our choice to correct the population structure in the GWAS and the replication study by using the country of sampling instead of the genetic PCs as covariates. The two methods produce highly correlated test statistics and overlapping top association signals (see Material and Methods) but the approach of using country as a covariate is a correction method that can be also used in the replication stage where only candidate SNPs are genotyped and therefore genetic PCs cannot be calculated.

### GWAS

**Figure 5:**
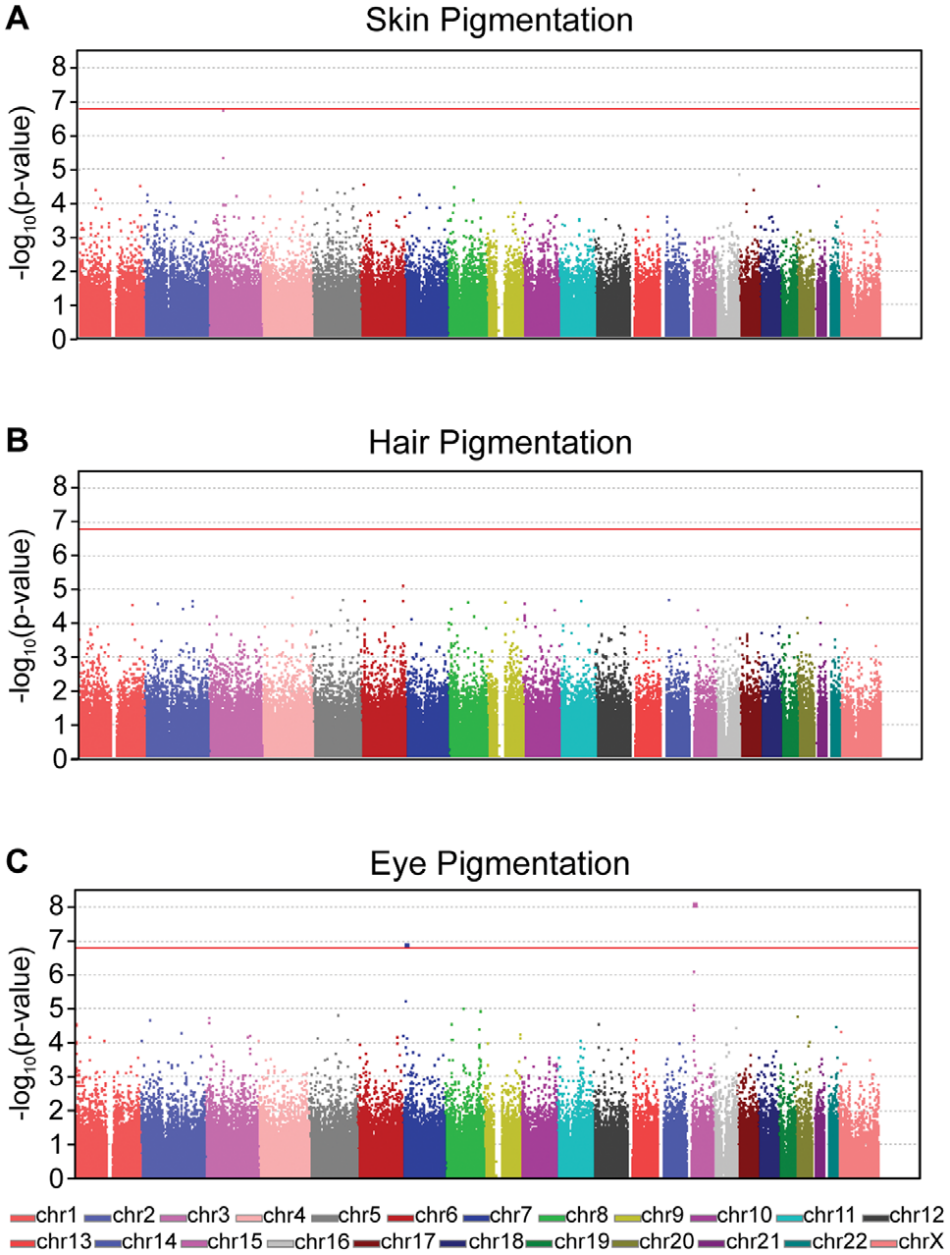
GWAS results. Manhattan plots for the GWAS results for the skin (**A**), hair (**B**), and eye (**C**) pigmentation. The log-transformed p-values from the test of association are plotted as a function of the chromosomal position. Genome-wide significance is defined as the Bonferroni corrected 5% significance threshold (p-value<1.6×10^−7^) and is indicated as a red line. For skin pigmentation, one SNP on chromosome 3 in the *FLNB* gene almost reaches genome-wide significance (p-value = 1.8×10^−7^). No SNP achieves genome-wide significance in the hair pigmentation GWAS. For eye pigmentation, two SNPs, one near the pigmentation gene *OCA2* on chromosome 15 and one in the *SCIN* gene on chromosome 7 achieve genome-wide significance.

The association between the three quantitative pigmentation phenotypes and the genotypes at 313,763 SNPs was tested in 176 women (Figure 5). Phenotypic information was available for 175, 106, and 175 women for the skin, hair, and eye pigmentation GWAS, respectively. To account for multiple testing, the Bonferroni correction was applied and the genome-wide 5% significance threshold was considered to be a p-value <1.6×10^−7^ (0.05/313,763). At this significance level, two SNPs are associated with eye pigmentation: rs1667394 (p = 8.1×10^−9^) and rs702477 (p = 1.3×10^−7^). No SNP was associated with hair and skin pigmentation, although one SNP, rs9809315, almost reaches genome-wide significance level for the association with skin pigmentation (p = 1.8×10^−7^) (Table 2 and Figure 5). rs1667394 is located in an intron of *HERC2* near the *OCA2* gene, the major locus determining eye color and this SNP has been reproducibly associated with eye color [13]. rs702477 is located in an intron of scinderin (*SCIN*) and rs9809315 is located in an intron of filamin B (*FLNB*). To our knowledge, neither *SCIN* nor *FLNB* has been implicated in pigmentation.

**Table 2:** GWAS, replication, and combined association results for all signals with p-value<10^−5^ in the GWAS.

					GWAS			Replication			Combined		
Trait	SNP	Chr	Gene	Alleles*	AF†	Beta^‡^	p-value	AF	Beta	p-value	AF	Beta	p-value
Eye C'	rs1667394	15q13.1	*HERC2*	C/T	0.29	0.41	8.08E–09	0.27	0.33	2.96E–13	0.28	0.35	1.92E–20
Eye C'	rs702477	7p21.3	*SCIN*	C/T	0.34	0.34	1.30E–07	0.31	−0.02	6.88E–01	0.32	0.12	2.30E–03
Eye C'	rs8039195	15q13.1	*HERC2*	C/T	0.25	0.37	7.91E–07	0.26	0.35	3.66E–13	0.25	0.35	2.77E–18
Eye C'	rs886890	7p21.3	*SCIN*	T/C	0.48	−0.27	5.65E–06	0.53	0.03	4.77E–01	0.51	−0.09	1.01E–02
Eye C'	rs1635168	15q13.1	*HERC2*	T/G	0.15	0.41	7.36E–06	0.13	0.26	3.21E–05	0.14	0.31	1.17E–09
Skin M	rs9809315	3p14.3	*FLNB*	A/G	0.31	1.26	1.79E–07	0.30	0.34	9.36E–02	0.30	0.65	2.84E–05
Skin M	rs2033739	3p14.3	*FLNB*	A/G	0.36	1.10	4.57E–06	0.34	0.17	4.32E–01	0.35	0.52	1.41E–03
Hair M	rs262825	6q25.3	*GTF2H5, TULP4*	C/T	0.47	11.77	7.94E–06	0.47	0.89	5.35E–01	0.47	3.63	4.12E–03

* Minor/Major allele in the GWAS.

† Allele frequencies (AF) and ^‡^regression coefficients (beta) are given with respect to the number of copies of the minor allele in the GWAS.

For quality control, the genomic control inflation factor, lambda [23], was calculated and Q-Q plots were drawn. Lambda values are close to the expected value of 1. They are 1.00, 1.02, and 0.99 for the skin, hair, and eye GWAS, respectively. Q-Q plots show no obvious deviation from expectation (Figure S1). These results indicate no evidence for a systematic bias or inflation of our test statistics by, for example, uncorrected population stratification. In contrast, if the GWAS are performed without a correction for population structure, we see a large deviation from expectation for both lambda (1.15, 1.11, and 1.23 for skin, hair, and eye pigmentation) and the Q-Q plots (Figure S1). Interestingly, we found that in the absence of a correction for population structure, skin pigmentation shows a single very strong false positive association with a 1 Mb region on chromosome 2 that spans the lactase (*LCT*) gene (smallest p-value = 8.2×10^−13^ is for rs932206). The *LCT* gene is a locus well-documented as showing substantial genetic differentiation within Europe [29], [30]. Population structure acts as a confounder because both the lactase-persistence allele at *LCT* and lighter skin pigmentation are correlated with the North-South cline within Europe. This false positive association between the *LCT* locus and skin pigmentation mirrors the false positive association found between the *LCT* locus and height [31], [32]. For eye pigmentation, the presence of population structure results in inflated test statistics at the *OCA2/HERC2* locus (smallest p-value = 5.4×10^−17^ is for rs1667394). These results demonstrate that not accounting for European population structure, particularly in this situation where the phenotypes are stratified along axes of genetic differentiation, leads to highly significant false positive results (*LCT* locus for skin pigmentation) or inflated true positive results (*OCA2/HERC2* locus for eye pigmentation) (Figure S2).

### Replication

Because our method detects the strong genetic effect of the *OCA2/HERC2* locus on eye pigmentation, it is reasonable to assume that our small sample size explains why our GWAS p-values for other causative loci are not clearly smaller than those expected by chance. Thus we decided to use a liberal p-value cut-off in the GWAS to select candidate SNPs for a more comprehensive replication study. Twenty-six, 33, and 44 SNPs met the criteria for the skin, hair, and eye pigmentation GWAS, respectively. Individuals included in the replication had ancestry in the same four European countries, Ireland, Poland, Italy, and Portugal, as in the GWAS. To avoid confounding by population structure at the replication stage, we only considered 294 individuals (153 males and 141 females), who reported that all of their four grandparents had ancestry in the countries of sampling. Quantitative phenotypes were available for 294, 235, and 293 of these individuals for the skin, hair, and eye pigmentation, respectively.

Twenty-one, 26, and 34 of the candidate SNPs passed the genotyping quality control for the skin, hair, and eye pigmentation replication, respectively, and these include the top association signals of the three GWAS. GWAS, replication, and GWAS and replication combined results for the eight SNPs with p-value <10^−5^ in the GWAS are presented in Table 2, while results for the 103 SNPs with p-values <10^−4^ in the GWAS are presented in Table S3. Using a Bonferroni correction per phenotype to set the significance threshold for the replication at 5% (p<2.4×10^−3^ for skin, p<1.9×10^−3^ for hair, and p<1.5×10^−3^ for eye pigmentation), we find that five SNPs at the *OCA2/HERC2* locus are reproducibly associated with eye pigmentation. Considering the number of loci for the Bonferroni correction (18, 24, and 26 for skin, hair and eye pigmentation) instead of the number of SNPs leads to a less conservative correction but to identical conclusions. Since the GWAS included only females, we also performed the analysis considering only the females and obtained qualitatively identical conclusions: no SNP is reproducibly associated with skin or hair pigmentation, while five SNPs at the *OCA2/HERC2* locus are reproducibly associated with eye pigmentation in the replication female only cohort.

These five SNPs are rs1667394, rs8039195, rs1635168, rs16950987, and rs8028689. They span a 46kb interval. rs1667394 is the most significant (p = 3.0×10^−13^ in the replication). It is located 186 kb 5′ of *OCA2* first exon and within an *HERC2* intron and is in linkage disequilibrium (LD) with the other four SNPs (r^2^ = 0.91, 0.46, 0.39, and 0.39 with rs8039195, rs1635168, rs16950987, and rs8028689, respectively in HapMap release 27 CEU). rs1667394 is also in LD with rs12913832 (D’ = 1, r^2^ = 0.65), which was not genotyped in our study but is thought to be the likely causative variant for blue versus brown eye color by genetic and functional analyses [33], [34]. The derived allele (G) at rs12913832 is recessive and is associated with blue eye color. The genotype at rs12913832 was imputed in the GWAS samples and the significance of the association with eye color, correcting for ancestry was found to be of similar magnitude at the imputed rs12913832 (p = 2.0×10^−12^) as at the genotyped rs1667394 (p = 5.8×10^−12^) considering that the derived allele is recessive. Adjusting for the recessive effect of the rs12913832 derived allele in the GWAS did not provide any new genome-wide significant signal. Furthermore the existing signal at rs1667394 was abolished (p = 0.73), indicating that rs1667394 and rs12913832 likely represent the same association signal. The derived allele frequency of the imputed rs12913832 is 0.90, 0.83, 0.59 and 0.44 and the genetic effect of rs12913832 explains 20%, 42%, 12%, and 33% of the variance in quantitative eye color in our GWAS sample from Ireland, Poland, Italy, and Portugal, respectively. The percent variance explained is greatest in Poland, where there is the largest variation in eye color.

## Discussion

In this study, we measured skin, hair, and eye pigmentation in four European countries and conducted a GWAS for each pigmentary trait. We used the quantitative measurements to analyze correlations between these pigmentary phenotypes and to test for stratification of the phenotypes along axes of European genetic variation. Objective and quantitative phenotyping has the potential to improve the power to detect a genetic effect compared to GWAS based on subjective categorical phenotypes, but due to small sample size our study was only sufficiently powered to identify the major genetic effect of the *HERC2/OCA2* locus on eye color. These results are consistent with the currently known genetic architecture of hair and eye color and allow us to make a prediction regarding the genetic architecture of skin pigmentation variation in Europe.

For eye color, we replicated the known and strong association of the *HERC2* SNP rs1667394. rs1667394 is in LD with rs12913832, a SNP that was not genotyped in our study but is the most strongly associated with and a likely causative variant for the blue eye color phenotype [33]–[35]. rs12913832 is a strong predictor of eye color; it alone explains 44–48% of the variance in quantitative measures of eye color in Dutch Europeans [11]. For such a strong genetic effect, the sample size of our GWAS has 100% power to detect the association at this locus. In contrast, other eye color loci reported [11] have been estimated each to explain less than 1% of the phenotypic variation, for which our GWAS sample size is under-powered. For hair color, we had 80% power to detect a genetic effect explaining 29% of the phenotypic variation. In a large GWAS based on subjective categorization of hair color [9], the strongest genetic effect detected was also at rs12913832. This genetic factor explains 10.7% of the blond to black variation in hair color in populations of European ancestry, requiring a sample size of 327 to achieve 80% power. For the spectroscopic measurement of skin pigmentation, we had 80% power to detect an effect that explains 19% of the variance in the phenotype. The fact that we did not detect reproducible associations with skin or hair color suggests that, unlike eye color, skin and hair pigmentation variation in Europe are not determined by major loci.

The skin pigmentation phenotype was of particular interest in our study since it is a continuously distributed phenotype that, to our knowledge, has not been the subject of a genome-wide scan in Europeans using an objectively quantified measure of skin color. However our results can be compared to the Stokowski et al. [17] GWAS, which showed that non-synonymous variants in three genes, rs1426654 *SLC24A5*, rs16891982 *SLC45A2,* and rs1042602 *TYR,* contribute to differences in spectroscopic skin pigmentation measurements in South Asians. In candidate gene association studies, these three variants have been shown to contribute to quantified skin pigmentation variation in admixed European/African populations [4], [36]–[38]. In Europe, variation at rs1426654 in *SLC24A5* does not contribute to skin pigmentation differences since the derived allele associated with light skin pigmentation has been swept to fixation (100% frequency in HapMap CEU). rs16891982 in *SLC45A2* was not genotyped on our Illumina platform. This variant (98% frequency in CEU for the derived allele) may contribute to skin pigmentation differences in Europe as suggested in a candidate gene study for subjective skin color [39]. We also note that as a follow-up to a resequencing study of *SLC45A2* (NAG and GSB unpublished), we genotyped an *SLC45A2* intronic variant, r183671, in the replication cohort along with six other SNPs in pigmentation candidate genes. rs183671 is located 13 kb away from rs16891982 and is in LD with it in CEU (D’ = 1, r^2^ = 0.5). The frequency of the rs183671 derived allele increases from Southern to Northern Europe: it is 88%, 89%, 98%, and 97% in the Portuguese, Italian, Polish, and Irish cohorts, respectively. We found that this SNP shows some evidence of association with skin pigmentation (p = 6×10^−4^, n = 289), and that each copy of the derived allele lightens the skin by 1.2 M index units, further arguing for an effect of *SLC45A2* on European skin pigmentation variation. In contrast to the rare SNPs in *SLC24A5* and *SLC45A2*, rs1042602 in *TYR* is highly polymorphic in Europe (43% frequency for the derived allele in CEU, 37% frequency in our GWAS). It is associated with freckles in Europeans [13] but we do not find evidence for its association with skin pigmentation in our study (p = 0.3). Lastly we note that the third most significant SNP in the skin pigmentation GWAS is the rare non-synonymous variant R163Q (rs885479, allele frequency of 3% in our GWAS, p = 1×10^−5^, Table S3) in the *MC1R* gene. *MC1R* is an extensively studied human pigmentation gene whose variants have been associated with red hair, fair skin, sun sensitivity, and freckling in populations of European ancestry (see [40] for a review). In candidate gene studies, R163Q has shown either no association, or association of the minor, derived allele (Q variant) with lighter skin pigmentation [41]–[45]. We did not obtain genotype data for this variant in the replication study, but we note that in our GWAS, unlike in previous candidate gene studies, the minor, derived allele is associated with darker skin pigmentation (Table S3). Studies including larger sample sizes are needed to characterize the R163Q variant effect on skin pigmentation.

The quantitative measurements of skin, hair, and eye pigmentation also provide some insight into the relationships among the phenotypes and their differentiation between sexes. We found that all three pigmentary phenotypes are correlated with each other even after correcting for population structure. This correlation may result from the existence of shared or linked genetic determinants of multiple pigmentation phenotypes, such as rs12913832 in *HERC2*, which was shown to be very strongly associated with eye color but more weakly associated with hair color [9], [34]. The correlation between phenotypes that we observe raises the question of whether the association of a variant with two pigmentary traits reflects a pleiotropic genetic effect on two related phenotypes, or whether it is mediated by the correlation between the phenotypes due to residual cryptic population stratification. Having measured multiple phenotypes on the same set of individuals we have an opportunity to address this question. One way to distinguish between the two possibilities is to determine if the association of rs12913832 with hair color holds after correcting for eye color. The idea here is that phenotypic association due to stratification will be removed by this correction, while true functional associations will not be completely removed. In our GWAS, the p-value for the association of hair pigmentation with imputed rs12913832 is 0.01. After correcting for eye color, the p-value is 0.02. This argues in favor of a model in which rs12913832 has a pleiotropic effect on both eye and hair color.

Interestingly, our analysis of variation in skin color in Europe demonstrates a consistent difference in skin color between the sexes. By the DermaSpectrometer M index measure, males are more lightly pigmented than females in each of the four European countries we studied. The same trend in M index was reported previously in a sample of European Americans [38]. Our results in populations of European ancestry contradict earlier anthropological studies that have concluded females are more lightly pigmented than males in most populations (reviewed in [2]). One potential reason for the conflicting results is the different instruments used. In early studies, which used the Evans Electric Limited (EEL) and Photovolt broad-spectrum spectrophotometers, skin pigmentation estimates may be confounded by the hemoglobin level to a greater extent than for the DermaSpectrometer used in the present study [46]. Lastly, we emphasize that differences in skin pigmentation between sexes are likely population specific. Whereas DermaSpectrometer M index measurements in this study show European males to have lighter skin pigmentation than European females, DermaSpectrometer M index measurements of skin pigmentation in Island Melanesian, African Caribbeans, and African Americans have shown that in these populations males have darker skin pigmentation than females [6], [38], [47].

While our study does not identify novel genetic determinants of pigmentation, it does demonstrate the extensive stratification of pigmentary phenotypes in Europe along the major axes of genetic variation. Because of this differentiation, any genetic analysis of pigmentary traits in Europe or in populations of European descent, such as European Americans, must carefully correct for population structure. This correction has been rigorously applied in GWAS studies by correcting for genetic PCs derived from whole-genome genotypic information. Previous studies have shown that more than 1,000 ancestry-informative markers are needed to derive the first two genetic PCs of European structure adequately representing the North/South and East/West axes of differentiation [32], [48]. This information is not usually available in candidate gene studies, in replication studies of GWAS, or in fine mapping studies. Not correcting for ancestry leads to an overestimation of the effect of the pigmentation genes, like the *HERC2/OCA2* locus for eye color, or results in a false positive association of loci that have differentiated along the same geographical axes, *e.g.* the strong association of variation at the lactase gene (*LCT)* with skin color that we observed. We also expect that the high levels of stratification for pigmentary phenotypes in Europe will lead to false negative findings due to the unaccounted effect of a strong confounder. In our GWAS, we have found that a correction of the association test based on country of origin performs equivalently to a correction using genetic PCs. Although the country based correction proved adequate in this particular instance, it is important to remember that this selection of four distinct European population samples from the corners of the continent and our exclusion of persons with known ancestry outside these countries, are expected to align the PC-based and country-based ancestry scores. One should be careful about generalizing this finding to other more heterogeneous sample like larger pan-European and European-American samples.

## Supporting Information

Figure S1 **Q-Q plots for the GWAS.** Q-Q plots of the observed p-values against the expected p-values drawn from a uniform distribution under the null hypothesis of no association. Results from the GWAS corrected (black) and uncorrected (gray) for population structure are shown for skin (**A**), hair (**B**), and eye pigmentation (**C**). The diagonal is indicated as a black dashed line and the 95% confidence interval is indicated between the gray dashed lines. The uncorrected p-values show an early deviation from expectation (at low –log p-value), indicative of the inflation in test statistics caused by European population structure. The p-values obtained after correcting for population structure do not deviate from the diagonal except in the last three points for the eye pigmentation GWAS, the more likely true positive results.
(TIF)

Figure S2 **GWAS results without correcting for population structure.** Manhattan plots of the GWAS results for the skin (**A**), hair (**B**), and eye (**C**) pigmentation with no correction for population structure. The log-transformed p-value from the test of association is plotted as a function of the chromosomal position. Genome-wide significance is defined as the Bonferroni corrected 5% significance threshold (p<1.6×10^−7^) and is indicated as a red line. For skin pigmentation, SNPs spanning a 1 Mb region on chromosome 2 that encompasses the lactase (*LCT*) gene are significantly associated (smallest p-value is 8.2×10^−13^ for rs932206). For hair pigmentation, one SNP, rs10868841, on chromosome 9q21, is significantly associated (p = 5.5×10^−8^). For eye pigmentation, SNPs in a 200kb interval at the *OCA2/HERC2* locus were significant (most significant was rs1667394, p = 5.4×10^−17^). Also two SNPs on chromosome 12p12 were significant (most significant was rs11046263 p = 3.9×10^−10^) and one SNP on chromosome 9q34 (rs10793902, p = 1.5×10^−7^) was significant.
(TIF)

Table S1 **Differences in pigmentation between countries and sexes (p-values from a linear model).**
(PDF)

Table S2 **Correlations between phenotypes (Pearson correlations and p-values from a linear model in parentheses).**
(PDF)

Table S3 **GWAS, replication, and combined association results for all signals with p-value<10** ^−**4**^ **in the GWAS.**
(PDF)
