# Supplementary figures and images for: Genome-Wide Association Studies of Quantitatively Measured Skin, Hair, and Eye Pigmentation in Four European Populations
Source: PLoS One. 2012 Oct 31;7(10):e48294. doi: 10.1371/journal.pone.0048294 (PMC3485197; doi:10.1371/journal.pone.0048294)

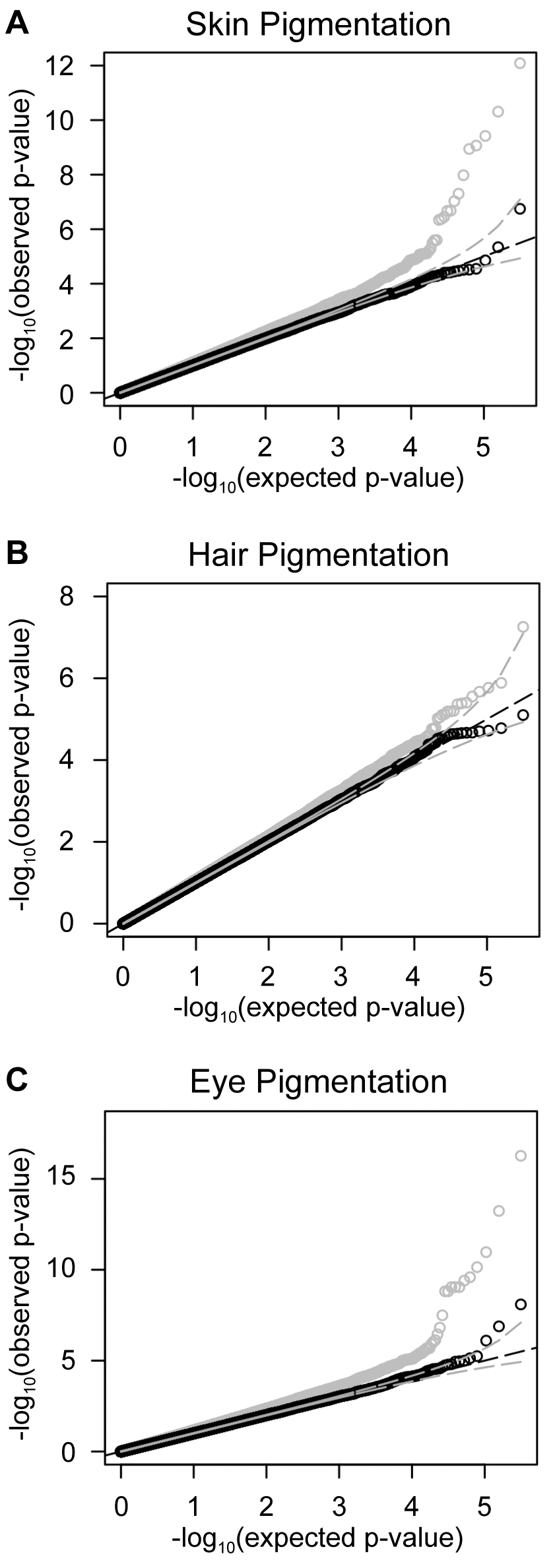

Supplement: Figure S1 — Q-Q plots for the GWAS. Q-Q plots of the observed p-values against the expected p-values drawn from a uniform distribution under the null hypothesis of no association. Results from the GWAS corrected (black) and uncorrected (gray) for population structure are shown for skin (A), hair (B), and eye pigmentation (C). The diagonal is indicated as a black dashed line and the 95% confidence interval is indicated between the gray dashed lines. The uncorrected p-values show an early deviation from expectation (at low –log p-value), indicative of the inflation in test statistics caused by European population structure. The p-values obtained after correcting for population structure do not deviate from the diagonal except in the last three points for the eye pigmentation GWAS, the more likely true positive results. (TIF) [file pone.0048294.s001.tif]

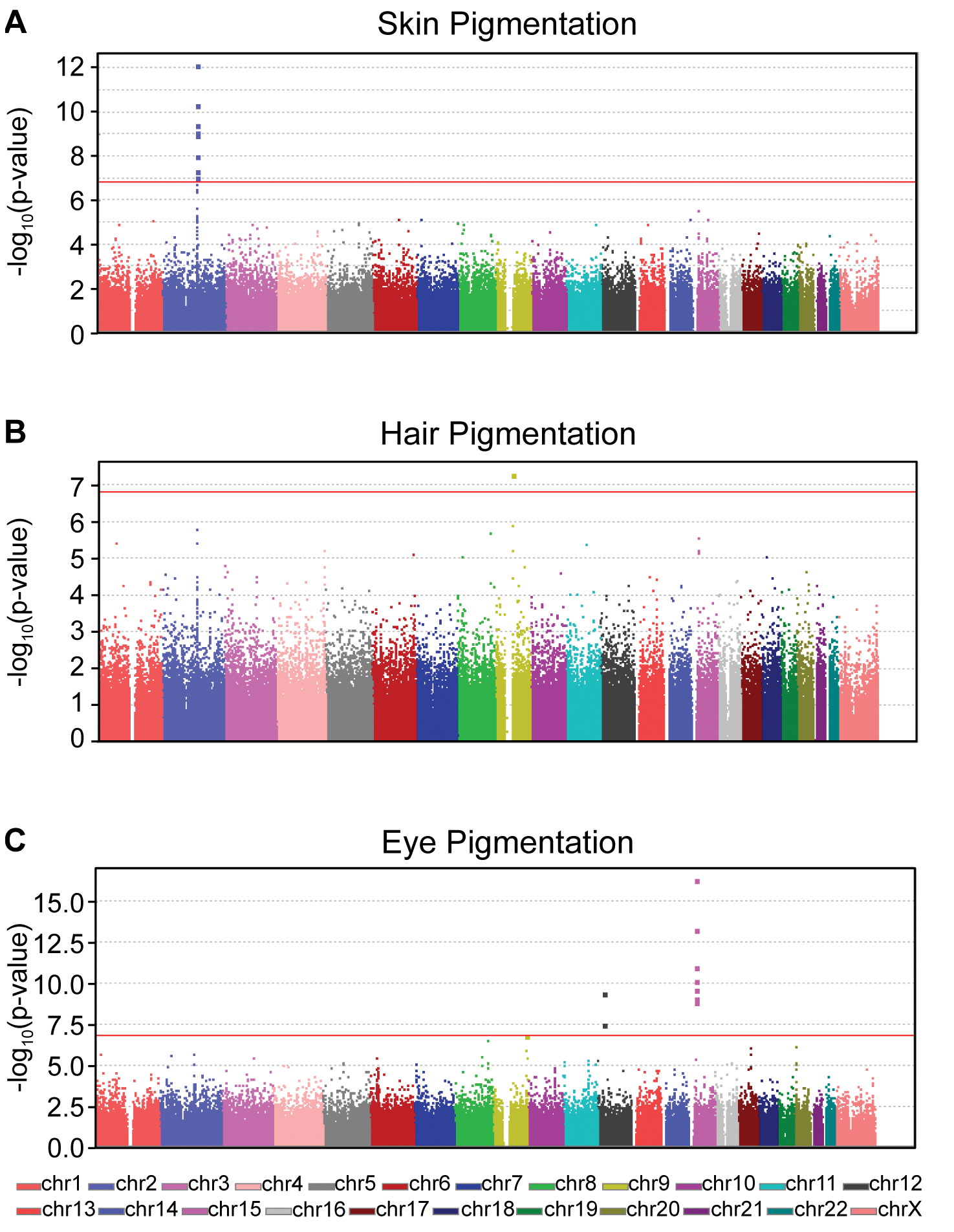

Supplement: Figure S2 — GWAS results without correcting for population structure. Manhattan plots of the GWAS results for the skin (A), hair (B), and eye (C) pigmentation with no correction for population structure. The log-transformed p-value from the test of association is plotted as a function of the chromosomal position. Genome-wide significance is defined as the Bonferroni corrected 5% significance threshold (p<1.6×10^−7^) and is indicated as a red line. For skin pigmentation, SNPs spanning a 1 Mb region on chromosome 2 that encompasses the lactase (LCT) gene are significantly associated (smallest p-value is 8.2×10^−13^ for rs932206). For hair pigmentation, one SNP, rs10868841, on chromosome 9q21, is significantly associated (p = 5.5×10^−8^). For eye pigmentation, SNPs in a 200kb interval at the OCA2/HERC2 locus were significant (most significant was rs1667394, p = 5.4×10^−17^). Also two SNPs on chromosome 12p12 were significant (most significant was rs11046263 p = 3.9×10^−10^) and one SNP on chromosome 9q34 (rs10793902, p = 1.5×10^−7^) was significant. (TIF) [file pone.0048294.s002.tif]
