# Supplementary material for: Genome-Wide Association Studies of Quantitatively Measured Skin, Hair, and Eye Pigmentation in Four European Populations
Source: PLoS One. 2012 Oct 31;7(10):e48294. doi: 10.1371/journal.pone.0048294 (PMC3485197; doi:10.1371/journal.pone.0048294)
Supplement: Table S1 — Differences in pigmentation between countries and sexes (p-values from a linear model). (PDF) [file pone.0048294.s003.pdf]

Table S1

		Country*			Sex
		Poland	Italy	Portugal	
Skin M (n=469)	Ireland	**<2E-16**	**<2E-16**	**<2E-16**	**8.E-04**
	Poland		**1.E-03**	0.67	**3.E-04**
	Italy			**1.E-04**	**6.E-03**
	Portugal				**2.E-05**
Hair M (n=341)	Ireland	**4.E-02**	**<2E-16**	**<2E-16**	**8.E-03**
	Poland		**9.E-10**	**4.E-08**	0.72
	Italy			**2.E-02**	0.30
	Portugal				0.46
Eye C' (n=468)	Ireland	**8.E-04**	**<2E-16**	**<2E-16**	0.83
	Poland		**4.E-10**	**<2E-16**	0.71
	Italy			0.17	0.16
	Portugal				0.16

*The linear model to test the effect of country included sex as a covariate.
In bold are p-values<0.05.
