# Supplementary material for: Genome-Wide Association Studies of Quantitatively Measured Skin, Hair, and Eye Pigmentation in Four European Populations
Source: PLoS One. 2012 Oct 31;7(10):e48294. doi: 10.1371/journal.pone.0048294 (PMC3485197; doi:10.1371/journal.pone.0048294)
Supplement: Table S2 — Correlations between phenotypes (Pearson correlations and p-values from a linear model in parentheses). (PDF) [file pone.0048294.s004.pdf]

Table S2

Phenotypes	Skin M, Eye C'	Eye C', Hair M	Skin M, Hair M
Ireland	0.18 (**0.027**)	0.10 (0.311)	0.11 (0.273)
Poland	0.01 (0.918)	-0.06 (0.664)	0.45 (**0.002**)
Italy	0.19 (0.051)	0.20 (0.090)	0.28 (**0.015**)
Portugal	0.12 (0.145)	0.37 (**3E-05**)	0.16 (0.090)
