# Supplementary material for: Genome-Wide Association Studies of Quantitatively Measured Skin, Hair, and Eye Pigmentation in Four European Populations
Source: PLoS One. 2012 Oct 31;7(10):e48294. doi: 10.1371/journal.pone.0048294 (PMC3485197; doi:10.1371/journal.pone.0048294)
Supplement: Table S3 — GWAS, replication, and combined association results for all signals with p-value<10 −4 in the GWAS. (PDF) [file pone.0048294.s005.pdf]

Table S3

						GWAS				Replication				Combined			
**Trait**	**SNP**	**Chr**	**Position (hg19)**	**Gene(s)**	**Alleles***	**AF†**	**Beta‡**	**SE**	**p-value**	**AF**	**Beta**	**SE**	**p-value**	**AF**	**Beta**	**SE**	**p-value**
Eye C'	rs1667394	15	28530182	HERC2	C/T	0.29	0.410	0.068	8.08E-09	0.27	0.334	0.044	2.96E-13	0.28	0.353	0.036	1.92E-20
Eye C'	rs702477	7	12660526	SCIN	C/T	0.34	0.342	0.062	1.30E-07	0.31	-0.019	0.048	6.88E-01	0.32	0.118	0.038	2.30E-03
Eye C'	rs8039195	15	28516084	HERC2	C/T	0.25	0.365	0.071	7.91E-07	0.26	0.352	0.046	3.66E-13	0.25	0.351	0.039	2.77E-18
Eye C'	rs886890	7	12665739	SCIN	T/C	0.48	-0.268	0.057	5.65E-06	0.53	0.033	0.047	4.77E-01	0.51	-0.095	0.037	1.01E-02
Eye C'	rs1635168	15	28535266	HERC2	T/G	0.15	0.408	0.088	7.36E-06	0.13	0.262	0.062	3.21E-05	0.14	0.312	0.050	1.17E-09
Eye C'	rs7834766	8	68793432	CPA6 PREX2	T/C	0.46	-0.277	0.061	1.00E-05	0.45	0.027	0.044	5.46E-01	0.45	-0.083	0.036	2.31E-02
Eye C'	rs4778241	15	28338713	OCA2	T/G	0.28	0.308	0.068	1.07E-05	NA	NA	NA	NA	NA	NA	NA	NA
Eye C'	rs4596632	8	132353735	ADCY8 EFR3A	C/T	0.39	0.269	0.059	1.14E-05	0.30	0.104	0.044	2.04E-02	0.33	0.167	0.035	2.96E-06
Eye C'	rs288139	5	107400505	FBXL17	A/G	0.22	0.321	0.072	1.57E-05	0.20	0.140	0.051	6.49E-03	0.21	0.206	0.042	1.01E-06
Eye C'	rs6056505	20	9243782	PLCB4	T/C	0.06	-0.547	0.123	1.64E-05	0.07	0.107	0.083	1.99E-01	0.07	-0.116	0.070	9.61E-02
Eye C'	rs10865738	3	16133383	GALNTL2	G/A	0.38	-0.281	0.064	1.81E-05	0.34	0.020	0.043	6.42E-01	0.36	-0.081	0.036	2.61E-02
Eye C'	rs2048983	2	35916128	MYADML CRIM1	C/T	0.33	-0.280	0.064	2.21E-05	0.32	0.063	0.047	1.78E-01	0.32	-0.067	0.038	8.13E-02
Eye C'	rs9830122	3	16127222	GALNTL2	C/T	0.38	-0.274	0.063	2.67E-05	0.39	0.045	0.045	3.15E-01	0.39	-0.069	0.037	6.39E-02
Eye C'	rs11046263	12	22187791	ABCC9 CMAS	C/T	0.18	-0.336	0.078	2.77E-05	0.25	0.002	0.050	9.66E-01	0.22	-0.108	0.043	1.12E-02
Eye C'	rs12546464	8	22627471	PEBP4	C/T	0.17	0.337	0.078	2.91E-05	0.24	0.034	0.048	4.74E-01	0.22	0.117	0.041	4.51E-03
Eye C'	rs1725265	1	7558193	CAMTA1	C/T	0.48	0.248	0.058	2.91E-05	0.53	-0.003	0.043	9.45E-01	0.51	0.090	0.035	9.51E-03
Eye C'	rs12562147	1	7552619	CAMTA1	A/G	0.42	0.246	0.057	3.20E-05	0.46	-0.031	0.045	4.93E-01	0.44	0.080	0.036	2.53E-02
Eye C'	rs7511306	22	44700587	KIAA1644	T/C	0.22	0.296	0.070	3.46E-05	NA	NA	NA	NA	NA	NA	NA	NA
Eye C'	rs305071	16	85949271	IRF8	T/C	0.10	-0.405	0.095	3.64E-05	NA	NA	NA	NA	NA	NA	NA	NA
Eye C'	rs2720672	8	129117936	PVT1 LOC728724	C/T	0.48	-0.262	0.062	4.01E-05	0.57	-0.043	0.042	3.02E-01	0.54	-0.119	0.035	6.71E-04
Eye C'	rs6527867	X	13653353	EGFL6 TCEANC	A/C	0.47	-0.244	0.059	4.94E-05	NA	NA	NA	NA	NA	NA	NA	NA
Eye C'	rs7558722	2	154698834	RPRM GALNT13	A/G	0.33	-0.265	0.064	5.02E-05	0.37	0.075	0.046	1.01E-01	0.36	-0.054	0.037	1.53E-01
Eye C'	rs10124840	9	138616058	KCNT1	C/T	0.17	-0.344	0.083	5.49E-05	NA	NA	NA	NA	NA	NA	NA	NA
Eye C'	rs7799050	7	4539599	SDK1 FOXK1	T/G	0.43	0.266	0.065	6.15E-05	0.43	0.063	0.043	1.41E-01	0.43	0.127	0.036	4.04E-04
Eye C'	rs6768086	3	166074779	BCHE ZBBX	G/A	0.23	-0.312	0.076	6.20E-05	0.24	0.017	0.050	7.31E-01	0.24	-0.087	0.042	3.82E-02
Eye C'	rs7495174	15	28344238	OCA2	C/T	0.15	0.342	0.083	6.22E-05	NA	NA	NA	NA	NA	NA	NA	NA

Eye C'	rs3773786	3	159610911	SCHIP1	A/G	0.25	-0.301	0.074	6.73E-05	0.23	0.024	0.052	6.46E-01	0.24	-0.092	0.043	3.15E-02
Eye C'	rs237012	6	149729198	TAB2	C/T	0.43	-0.247	0.061	6.98E-05	0.47	-0.006	0.046	9.02E-01	0.45	-0.100	0.037	6.82E-03
Eye C'	rs3005884	1	56771709	USP24<br>PPAP2B	C/T	0.35	0.246	0.060	7.01E-05	0.35	0.049	0.048	3.07E-01	0.35	0.128	0.037	6.41E-04
Eye C'	rs10940981	5	31938458	PDZD2	C/T	0.21	-0.291	0.071	7.06E-05	0.23	-0.041	0.053	4.38E-01	0.23	-0.135	0.043	1.69E-03
Eye C'	rs17168654	7	15124747	DGKB<br>AGMO	A/G	0.04	0.620	0.153	7.46E-05	0.07	-0.042	0.082	6.07E-01	0.06	0.116	0.074	1.15E-01
Eye C'	rs10448312	9	137900695	FCN1<br>OLFM1	G/A	0.39	-0.255	0.063	7.67E-05	NA	NA	NA	NA	NA	NA	NA	NA
Eye C'	rs7709159	5	147675836	SPINK13<br>SPINK7	A/G	0.01	-1.148	0.284	7.91E-05	0.02	0.056	0.149	7.09E-01	0.02	-0.238	0.135	7.76E-02
Eye C'	rs2553244	8	31042409	WRN<br>NRG1	A/G	0.48	-0.236	0.058	8.00E-05	0.50	0.050	0.041	2.27E-01	0.49	-0.053	0.034	1.19E-01
Eye C'	rs10105359	8	22632055	PEBP4	C/A	0.18	0.314	0.078	8.03E-05	NA	NA	NA	NA	NA	NA	NA	NA
Eye C'	rs16950987	15	28526228	HERC2	A/G	0.14	0.363	0.090	8.10E-05	0.13	0.274	0.065	3.73E-05	0.13	0.305	0.052	1.12E-08
Eye C'	rs8028689	15	28488888	HERC2	G/A	0.14	0.363	0.090	8.10E-05	0.11	0.250	0.070	3.99E-04	0.12	0.292	0.054	1.11E-07
Eye C'	rs9591586	13	33777482	STARD13	T/G	0.12	-0.367	0.091	8.20E-05	0.11	-0.045	0.071	5.24E-01	0.11	-0.175	0.056	1.87E-03
Eye C'	rs896918	2	5355848	LOC727982<br>SOX11	C/T	0.42	-0.248	0.062	8.55E-05	NA	NA	NA	NA	NA	NA	NA	NA
Eye C'	rs2274232	1	111773600	CHI3L2	C/T	0.07	-0.478	0.119	8.65E-05	0.07	0.101	0.079	2.05E-01	0.07	-0.094	0.067	1.62E-01
Eye C'	rs501234	11	87829246	TMEM135<br>RAB38	A/G	0.06	-0.523	0.130	9.03E-05	0.03	-0.133	0.129	3.05E-01	0.04	-0.336	0.091	2.46E-04
Eye C'	rs875130	4	2402706	ZFYVE28	G/A	0.26	0.255	0.064	9.13E-05	0.32	-0.006	0.044	8.94E-01	0.30	0.084	0.037	2.17E-02
Eye C'	rs1193180	1	7568982	CAMTA1	T/G	0.32	-0.255	0.064	9.17E-05	0.35	0.016	0.048	7.38E-01	0.34	-0.092	0.039	1.74E-02
Eye C'	rs2426638	20	55287567	TFAP<br>BMP7	C/T	0.26	-0.296	0.074	9.69E-05	NA	NA	NA	NA	NA	NA	NA	NA
Hair M	rs262825	6	158678631	GTF2H5<br>TULP4	C/T	0.47	11.770	2.499	7.94E-06	0.47	0.886	1.427	5.35E-01	0.47	3.631	1.257	4.12E-03
Hair M	rs7688185	4	116779145	NDST4<br>TRAM1L1	A/C	0.18	-13.870	3.066	1.66E-05	0.19	-0.451	1.984	8.20E-01	0.19	-4.534	1.675	7.14E-03
Hair M	rs4559841	14	28352815	LOC100505967<br>FOXG1	T/C	0.31	11.520	2.567	1.91E-05	0.35	1.018	1.800	5.72E-01	0.33	4.401	1.499	3.56E-03
Hair M	rs7712713	5	111900828	FLJ11235<br>APC	T/C	0.23	11.150	2.491	1.99E-05	0.26	-1.021	1.830	5.77E-01	0.24	3.194	1.512	3.53E-02
Hair M	rs13195345	6	15364430	JARID2	A/G	0.25	-11.260	2.525	2.13E-05	0.28	0.948	1.701	5.78E-01	0.27	-2.857	1.441	4.82E-02
Hair M	rs12475038	2	184037893	NUP35<br>ZNF804A	C/T	0.23	11.740	2.633	2.15E-05	0.20	1.888	1.701	2.68E-01	0.21	4.748	1.447	1.14E-03
Hair M	rs294877	6	159606873	FNDC1	C/T	0.38	-10.030	2.253	2.19E-05	0.28	4.491	1.784	1.25E-02	0.32	-0.512	1.430	7.21E-01
Hair M	rs1790708	11	82331434	ODZ4<br>FAM181B	T/C	0.05	-23.880	5.373	2.26E-05	NA	NA	NA	NA	NA	NA	NA	NA
Hair M	rs1594755	8	75845091	PI15<br>CRISPLD1	C/T	0.10	-23.050	5.167	2.30E-05	NA	NA	NA	NA	NA	NA	NA	NA
Hair M	rs10868841	9	73118664	KLF9<br>TRPM3	T/G	0.32	-11.720	2.642	2.33E-05	0.34	-0.257	1.735	8.82E-01	0.33	-3.646	1.477	1.40E-02

Hair M	rs4442466	10	1803525	ADARB2 LOC282980	C/T	0.39	-11.510	2.609	2.56E-05	NA	NA	NA	NA	NA	NA	NA	NA
Hair M	rs7594170	2	51237767	NRXN1	G/A	0.47	-10.860	2.462	2.60E-05	NA	NA	NA	NA	NA	NA	NA	NA
Hair M	rs2275697	1	205027737	CNTN2	A/G	0.13	-15.630	3.559	2.79E-05	0.19	0.712	1.853	7.01E-01	0.17	-2.549	1.676	1.29E-01
Hair M	rs1444715	X	25777511	ARX MAGEB18	C/T	0.43	-9.670	2.208	2.90E-05	NA	NA	NA	NA	NA	NA	NA	NA
Hair M	rs2675098	2	184005606	NUP35	G/A	0.23	11.890	2.722	3.03E-05	0.19	2.464	1.809	1.74E-01	0.20	5.375	1.519	4.59E-04
Hair M	rs4307347	8	10168094	MSRA	T/C	0.19	-13.930	3.230	3.74E-05	0.14	3.347	2.151	1.21E-01	0.16	-2.030	1.830	2.68E-01
Hair M	rs6712291	2	143550868	LRP1B KYNU	C/T	0.12	-14.540	3.365	3.78E-05	0.11	-2.428	2.624	3.56E-01	0.11	-7.318	2.089	5.25E-04
Hair M	rs11197567	10	117932193	GFRA1	T/C	0.23	-12.020	2.798	4.02E-05	0.25	-3.186	1.719	6.53E-02	0.24	-5.325	1.488	3.99E-04
Hair M	rs16959551	15	35004797	GOLGA8B GJD2	T/C	0.06	-20.350	4.748	4.15E-05	0.06	0.798	3.138	8.00E-01	0.06	-5.626	2.670	3.58E-02
Hair M	rs17160255	5	106951357	EFNA5	A/G	0.21	-11.380	2.656	4.17E-05	NA	NA	NA	NA	NA	NA	NA	NA
Hair M	rs17160261 (surrogate for rs17160255)	5	106954545	EFNA5	T/A	NA	NA	NA	NA	0.21	-0.620	1.956	7.51E-01	NA	NA	NA	NA
Hair M	rs7080882	10	2864447	LOC399708 PFKP	G/A	0.34	-9.504	2.260	5.63E-05	0.40	-2.732	1.589	8.70E-02	0.38	-4.946	1.294	1.57E-04
Hair M	rs2513852	8	99008727	MATN2	C/A	0.24	-11.800	2.814	5.91E-05	0.25	-2.240	1.827	2.21E-01	0.25	-5.328	1.547	6.45E-04
Hair M	rs4955192	3	31634537	STT3B	G/A	0.38	10.440	2.496	6.11E-05	0.41	-1.280	1.731	4.60E-01	0.40	2.845	1.446	5.00E-02
Hair M	rs4812405	20	35276585	SLA2 NDRG3	T/G	0.06	-21.980	5.279	6.61E-05	NA	NA	NA	NA	NA	NA	NA	NA
Hair M	rs6560768	10	1799598	ADARB2 LOC282980	G/T	0.44	-11.260	2.707	6.71E-05	0.45	0.873	1.641	5.95E-01	0.44	-2.299	1.437	1.11E-01
Hair M	rs1536328	9	116514876	RGS3 ZNF618	C/T	0.36	9.815	2.368	7.10E-05	0.45	-1.190	1.568	4.49E-01	0.41	2.190	1.334	1.02E-01
Hair M	rs2188866	7	20604449	ITGB8 ABCB5	T/C	0.21	14.420	3.471	7.11E-05	0.13	2.633	2.364	2.67E-01	0.16	6.340	1.904	9.69E-04
Hair M	rs9396529	6	14736650	CD83 JARID2	T/C	0.24	-12.240	2.958	7.38E-05	0.18	1.777	1.955	3.64E-01	0.20	-1.946	1.658	2.41E-01
Hair M	rs3750683	10	1779498	ADARB2	G/A	0.30	-10.740	2.609	7.84E-05	NA	NA	NA	NA	NA	NA	NA	NA
Hair M	rs250858	5	132716935	FSTL4	A/C	0.32	10.980	2.669	7.99E-05	0.33	-2.040	1.844	2.70E-01	0.32	2.049	1.555	1.89E-01
Hair M	rs997934	10	1795194	ADARB2 LOC282980	A/G	0.37	-10.310	2.511	8.20E-05	0.36	0.274	1.629	8.66E-01	0.36	-2.598	1.386	6.19E-02
Hair M	rs4461920	8	3285570	CSMD1	A/G	0.07	-18.530	4.532	9.15E-05	0.06	1.609	2.993	5.91E-01	0.07	-4.371	2.546	8.70E-02
Hair M	rs200708	21	24810659	D21S2088E LOC339622	C/T	0.26	-10.530	2.593	9.67E-05	0.31	2.480	1.841	1.79E-01	0.29	-1.910	1.528	2.12E-01
Skin M	rs9809315	3	58050265	FLNB	A/G	0.31	1.256	0.231	1.79E-07	0.30	0.339	0.201	9.36E-02	0.30	0.653	0.154	2.84E-05
Skin M	rs2033739	3	58063819	FLNB	A/G	0.36	1.103	0.233	4.57E-06	0.34	0.168	0.213	4.32E-01	0.35	0.516	0.161	1.41E-03
Skin M	rs885479	16	89986154	MC1R	T/C	0.03	2.711	0.606	1.39E-05	NA	NA	NA	NA	NA	NA	NA	NA
Skin M	rs12526812	6	12763839	PHACTR1	C/T	0.07	1.934	0.449	2.84E-05	0.06	0.394	0.405	3.31E-01	0.06	0.962	0.306	1.78E-03
Skin M	rs4027079	1	234344093	SLC35F3	C/T	0.48	1.000	0.234	3.06E-05	0.57	0.261	0.211	2.18E-01	0.53	0.502	0.159	1.68E-03

Skin M	rs4818568	21	21780178	TMPRSS15 LINC00320	A/G	0.12	-1.405	0.328	3.11E-05	0.17	0.091	0.238	7.02E-01	0.15	-0.349	0.193	7.19E-02
Skin M	rs11135712	8	23110503	CHMP7	A/G	0.39	0.915	0.215	3.36E-05	NA	NA	NA	NA	NA	NA	NA	NA
Skin M	rs962514	5	152694849	NMUR2 GRIA1	T/G	0.20	1.166	0.275	3.57E-05	0.17	0.110	0.245	6.54E-01	0.18	0.488	0.186	9.04E-03
Skin M	rs6664692	1	63195660	DOCK7 ATG4C	C/T	0.15	1.337	0.316	3.84E-05	0.21	0.118	0.245	6.31E-01	0.19	0.435	0.194	2.51E-02
Skin M	rs4360041	5	17005751	MYO10 LOC285696	A/C	0.32	-0.993	0.235	3.93E-05	0.30	-0.001	0.222	9.98E-01	0.31	-0.396	0.165	1.70E-02
Skin M	rs10512493	17	60177504	MED13 TBC1D3P2	C/T	0.45	0.861	0.204	3.95E-05	0.48	0.225	0.184	2.23E-01	0.47	0.458	0.139	1.04E-03
Skin M	rs3935679	5	97478311	RIOK2 RGMB	T/C	0.48	0.905	0.217	4.65E-05	NA	NA	NA	NA	NA	NA	NA	NA
Skin M	rs4691238	4	156963692	CTSO PDGFC	A/G	0.40	-0.893	0.214	4.76E-05	0.42	-0.169	0.192	3.80E-01	0.41	-0.464	0.145	1.45E-03
Skin M	rs6826026	4	156974697	CTSO PDGFC	T/C	0.32	0.959	0.231	5.25E-05	0.40	0.025	0.207	9.03E-01	0.37	0.366	0.156	1.94E-02
Skin M	rs7725801	5	121720013	SNCAIP	C/T	0.10	1.457	0.351	5.27E-05	0.07	-0.524	0.369	1.56E-01	0.08	0.375	0.263	1.55E-01
Skin M	rs10499703	7	52722167	COBL POM121L12	T/C	0.16	-1.267	0.306	5.50E-05	NA	NA	NA	NA	NA	NA	NA	NA
Skin M	rs871535	2	12746690	LPIN1 TRIB2	C/T	0.43	0.955	0.231	5.55E-05	0.51	-0.056	0.189	7.67E-01	0.48	0.260	0.147	7.75E-02
Skin M	rs1517605	3	106624294	LOC100302640	G/A	0.13	-1.353	0.329	6.01E-05	0.13	0.065	0.296	8.27E-01	0.13	-0.472	0.223	3.50E-02
Skin M	rs11934931	4	35183361	PCDH7 ARAP2	A/G	0.09	1.525	0.372	6.38E-05	0.11	0.080	0.307	7.95E-01	0.10	0.532	0.240	2.71E-02
Skin M	rs10499266	6	151572523	AKAP12	A/G	0.06	1.855	0.453	6.47E-05	0.04	1.017	0.504	4.45E-02	0.04	1.456	0.346	3.03E-05
Skin M	rs2225083	1	82960385	LPHN2 TTLL7	T/C	0.28	0.954	0.234	7.08E-05	0.28	0.242	0.209	2.47E-01	0.28	0.522	0.157	9.58E-04
Skin M	rs3102477	8	96746100	LOC100616530	T/G	0.02	3.173	0.786	8.12E-05	0.05	-0.528	0.415	2.04E-01	0.04	0.039	0.361	9.13E-01
Skin M	rs1505469	4	150848576	NR3C2 DCLK2	G/A	0.16	1.269	0.316	8.68E-05	0.12	-0.459	0.298	1.24E-01	0.14	0.241	0.223	2.81E-01
Skin M	rs2139381	2	12742090	LPIN1 TRIB2	T/C	0.49	0.907	0.226	8.69E-05	0.57	-0.072	0.189	7.04E-01	0.54	0.237	0.147	1.07E-01
Skin M	rs10760593	9	131953598	IER5L C9orf106	T/C	0.18	1.065	0.267	9.68E-05	0.20	-0.297	0.245	2.26E-01	0.19	0.223	0.185	2.28E-01
Skin M	rs3811561	2	101434684	PDCL3 NPAS2	A/C	0.20	1.109	0.278	9.91E-05	NA	NA	NA	NA	NA	NA	NA	NA

*Minor/Major allele in the GWAS.

†Allele frequencies (AF) and ‡regression coefficients (beta) are given with respect to the minor allele in the GWAS.

For SNPs that did not pass QC at replication or that were not genotyped in the GWAS, NA indicates no result.
